# Resting Heart Rate Variability Predicts Safety Learning and Fear Extinction in an Interoceptive Fear Conditioning Paradigm

**DOI:** 10.1371/journal.pone.0105054

**Published:** 2014-09-02

**Authors:** Meike Pappens, Mathias Schroijen, Stefan Sütterlin, Elyn Smets, Omer Van den Bergh, Julian F. Thayer, Ilse Van Diest

**Affiliations:** 1 Research Group on Health Psychology, KU Leuven, Leuven, Belgium; 2 Section of Psychology, Lillehammer University College, Lillehammer, Norway; 3 Department of Psychosomatic Medicine, Division of Surgery and Clinical Neuroscience, Oslo University Hospital - Rikshospitalet, Oslo, Norway; 4 Dept. of Psychology, Ohio State University, Columbia, Ohio, United States of America; Tokai University, Japan

## Abstract

This study aimed to investigate whether interindividual differences in autonomic inhibitory control predict safety learning and fear extinction in an interoceptive fear conditioning paradigm. Data from a previously reported study (N = 40) were extended (N = 17) and re-analyzed to test whether healthy participants' resting heart rate variability (HRV) - a proxy of cardiac vagal tone - predicts learning performance. The conditioned stimulus (CS) was a slight sensation of breathlessness induced by a flow resistor, the unconditioned stimulus (US) was an aversive short-lasting suffocation experience induced by a complete occlusion of the breathing circuitry. During acquisition, the paired group received 6 paired CS-US presentations; the control group received 6 explicitly unpaired CS-US presentations. In the extinction phase, both groups were exposed to 6 CS-only presentations. Measures included startle blink EMG, skin conductance responses (SCR) and US-expectancy ratings. Resting HRV significantly predicted the startle blink EMG learning curves both during acquisition and extinction. In the unpaired group, higher levels of HRV at rest predicted safety learning to the CS during acquisition. In the paired group, higher levels of HRV were associated with better extinction. Our findings suggest that the strength or integrity of prefrontal inhibitory mechanisms involved in safety- and extinction learning can be indexed by HRV at rest.

## Introduction

Fear conditioning research has yielded a wide array of laboratory models, tools and sophisticated experimental designs that are helpful to unravel the specific mechanisms that contribute to fear learning, and, potentially, to the pathogenesis of anxiety disorders [1], [2], [3]. In addition, fear conditioning research may substantially contribute to the identification of vulnerability factors for the development or maintenance of pathological fear. For example, it has been shown that anxiety patients are characterized by enhanced conditionability and fear generalization, by flattened extinction curves, by worse retention of extinction and by a reduced inhibition of fear responding to safety cues [3], [4]. Common to all these characteristics seems an impaired capacity to inhibit fear responding compared to healthy subjects. Therefore, interindividual differences in the capacity to inhibit fear responding may possibly represent a vulnerability factor for anxiety disorders.

Neuropsychological research has pointed to the crucial interaction of cortical and sub-cortical brain areas in the regulation of defensive behavior and its inhibition. Typically, the activation of medial subcortical areas underlying sympathetic-driven fear responding is regulated by top-down inhibitory input from the prefrontal cortex (PFC) [5], [6]. Successful fear extinction, for example, critically implies activation of the medial (m)PFC, a region that is anatomically densely connected to the amygdala [2], [5], [7]. Prefrontal areas are also involved in learning to discriminate between periods of safety and danger [8], [9].

A general indicator of prefrontal inhibitory capacity and adaptability to environmental changes may be found in vagally mediated heart rate variability (HRV) at rest, as put forward respectively in the model of neurovisceral integration (e.g. [10]–[12]) and the polyvagal theory [13]–[16]. Like most organs, the heart is dually innervated by the sympathetic and parasympathetic branches of the autonomic nervous system. Vagal activation tonically inhibits sympathetic modulation of heart rate. This process appears to be modulated via prefrontal inhibitory processes, affecting heart rate via cortico-cardiac pathways. These pathways have been described in detail elsewhere [11], [12]. Briefly, the dorsal mPFC (dmPFC) which is involved in threat responses and the ventral mPFC (vmPFC) which is more involved in antagonism of threat responses, modulate amygdala activity via GABAergic intercalated cells. The output of the amygdala via the nucleus of the solitary tract (NTS) impacts the output of the vagal motor neurons in the medulla through a network of interneurons connecting the NTS with the nucleus ambiguous (NA) and the dorsal motor nucleus of the vagus (DVM). The net effect is that sympatho-excitory circuits in the medulla are tonically inhibited by the vmPFC. Importantly, the output of this system can be indexed using HRV. Measured under resting conditions, the temporal stability of HRV is considered sufficiently high to justify the assumption that HRV is a stable individual difference variable [17], [18].

A wide variety of studies, comprising attentional processes [19], [20], memory retrieval [21], higher-order control processes involved in emotional decision-making [22] and emotional stability in everyday life [23], have documented significant relationships between these processes and vagally mediated resting HRV, suggesting that it indeed reflects a comprehensive measure of general inhibitory (emotional and/or behavioral) control capacity. Vagally mediated HRV at rest also predicts emotional adaptability in specific states and overall ability to regulate emotional responding [17], [24], [25]. For example, the relevance of resting HRV in the domain of defensive behavior is supported by both clinical and experimental data: anxiety disorder patients characterized by deficits in fear inhibition typically display low levels of HRV [11], [26], [27] and exaggerated fear-potentiated startles have been observed in persons with low resting HRV [27]–[29].

The close association between HRV and emotion regulation is further supported by neuroimaging studies showing that central nervous system correlates of HRV at rest substantially overlap with prefrontal areas relevant for emotion regulation and inhibitory control of subcortical, emotion-processing areas (for a meta-analysis see [12]).

If vagally mediated HRV may indeed serve as a proxy for prefrontal inhibitory control [11] it can be hypothesized that HRV should also be related to fear extinction and safety learning. Whereas several studies have already documented that vagally mediated HRV predicts individual differences in *fear response* magnitude [27]–[29], the question whether resting HRV modulates *fear extinction and safety learning* remains unanswered. Therefore, the current study aimed to investigate interindividual differences in HRV at rest as a predictor of safety learning and fear extinction success. To study this, we applied a recently developed interoceptive fear conditioning paradigm that aimed to establish fear or safety learning to an interoceptive CS [1]. In this paradigm, an ecologically relevant conditioned stimulus (CS, slight sensation of breathlessness) was paired with the occurrence of a panic-relevant unconditioned stimulus (US, suffocation experience) in one group (paired group), while the same CS signaled the absence of the same US in another (unpaired) group. Compared to more commonly used differential paradigms that use arbitrary CSs that are functionally unrelated to the US, the present paradigm is likely more relevant for fear learning to cardio-respiratory sensations that is assumed to occur in panic disorder patients. Using this interoceptive paradigm with a functionally related CS and US, no overall extinction of the fear potentiated startle was found in the paired group who received 100% reinforced CS-US pairings during acquisition. The unpaired (control) group for whom the CS technically spoken announced a ‘safe’ period, failed to display clear safety learning to the ecologically relevant CS. Because fear learning was overall strong, and complete fear extinction (paired group) or safety learning (unpaired group) were not established, the present paradigm may be a powerful one to study interindividual differences in inhibitory learning processes. We hypothesized that a higher cardiac vagal outflow would be associated with enhanced safety learning during acquisition in the unpaired group, and with improved fear extinction in the paired group.

## Method

### General Overview of Design, Stimuli and Measures

In this between subject paradigm, all participants received only one and the same CS: a slight sensation of breathlessness evoked by adding a flow resistor to the external breathing circuitry for 8 s. Such flow resistor slightly obstructs the air flow (increased resistance), requiring an increased respiratory muscle force to move the same amount of air into and out from the lungs, which feels similar to breathing through a straw. The US was a complete breathing obstruction (infinite resistance) during which participants could not breathe at all. During the US, the external breathing circuitry was occluded, impeding air of flowing into or out from the lungs. The length of the US was individually calibrated prior to the experiment and set at 40% of a participant's maximal breath holding time.

Both groups differed only with respect to when the US was administered relative to the CS during the fear acquisition phase. In the paired group, the CS was immediately followed by the US. Thus, the CS in the paired group signaled danger (the US). In the unpaired group, a relatively long inter stimulus interval (ISI) without any stimulation separated the CS and US in time. As such, the CS is technically speaking a relatively ‘safe’ period for the unpaired group. During the extinction phase, the US was not administered anymore, both groups received trials with only one CS.

Measures of fear learning included 1) fear potentiated startle responses (startle EMG), 2) skin conductance responses (SCRs) and 3) US expectancy ratings. Startle eyeblinks to acoustic startle probes were measured both during the CS and the ISI. As the eyeblink amplitude is potentiated during the anticipation of the US, startle responses are informative on how and when subcortical defensive motor preparation changes in relation to the experienced contingencies between the CS and the US. SCRs are generally considered to be sympathetically-mediated responses reflecting the novelty or relevance of a stimulus. US expectancy ratings are thought to represent declarative knowledge of the CS-US contingency.

Independent variables included the between-subject variables ‘Group’ (paired – unpaired), and heart rate variability (continuous predictor), and the within-subject variable ‘Block’ (1, 2, 3). For startle EMG, also ‘Probe’ (CS, ISI) was an additional within-subject variable.

### Ethics Statement

The experiment was approved by the Ethics Committee of the Department of Psychological and Educational Sciences of the University of Leuven and by the Medical Ethical Committee of the University Hospitals of the University of Leuven. Prior to the experiment, all subjects signed an informed consent form that was approved by the Ethics Committees of Psychology and Medical Sciences, stating – amongst other information – that participation was voluntary and that they could withdraw from the study at any moment.

### Participants

Fifty-seven healthy students (10 men, *M* = 22 years, range 18–30 years) participated in return for 10 €. Data on interoceptive conditioning effects on the forty participants who were run first have been reported elsewhere [1]. To increase the power to study interindividual differences in safety learning and extinction and the return of fear, the original dataset (N = 40, collected in 2009) was extended with another 17 participants (all women).

Participants did not suffer from any self-reported respiratory or cardiac diseases, epilepsy, psychiatric disorder, or any other minor or major illness, were nonsmokers and were not pregnant. Participants were randomly assigned to the paired (*N* = 29; 5 men) or the unpaired (*N* = 28; 5 men) group.

### Materials and Measures

Participants, wearing a nose-clip, breathed through a mouthpiece mounted on a non-rebreathing valve. Two vinyl tubes (3.5 cm diameter; 100 cm length) connected the inspiratory and expiratory side of the non-rebreathing valve with a 3-way (expiratory side) and a 4-way (inspiratory side) stopcock valve enabling easy switching between CS, US, and unloaded breathing. A nonaversive flow resistor of 10 cm H_2_O/l/s [30], [31] applied for 8 s or one complete breath served as the CS. This stimulus mildly restricts breathing and requires the participant to exert a bit more breathing effort to maintain normal ventilation. The US was a breathing obstruction (occlusion) applied for 40% of the individual's maximal postexpiratory breath-holding time (BHT) as determined prior to the conditioning procedure. For example, a BHT of 30 s resulted in a postexpiratory US of 12 s. The mean duration of the US (occlusion time) was 8.8 s (*SD* = 1.3 s). If BHT was less than 20 s, a minimum US of 8 s was administered.

All physiological signals were transmitted through a National Instruments card (12-bit A/D convertor) to a personal computer and stored using Affect 4.0 software [32].

The ECG was obtained using three standard Ag/AgCl electrodes (1 cm diameter) filled with electrolyte and placed on the thorax across the heart: two electrodes were placed below the left and right clavicle, one electrode was placed on the left lower rib cage. The signal was sampled at 1000 Hz and transduced, amplified and filtered through a Coulbourn S75-04 Isolated Bioamplifier. Low frequencies were cut off at 10 Hz, high frequencies at 1 kHz.

The startle eyeblink response was measured using Ag/AgCl Sensormedics electrodes (0.25 cm diameter) filled with electrolyte, by recording surface EMG activity over the *m. orbicularis oculi* just beneath the left eye [33]. The raw signal was amplified by a Coulbourn isolated bioamplifier with bandpass filter (V75-04; 13 Hz–1 kHz) and routed to a Coulbourn contour following integrator (S76-01), which rectified and smoothed the signal (time constant = 50 ms). Acoustic startle probes (95 dB, 50 ms duration) were administered binaurally.

Electrodermal activity (EDA) was recorded with Fukuda standard Ag/AgCl electrodes (1 cm diameter) filled with KY gel and attached to the hypothenar palm of the left hand, which was first cleaned with tap water. The interelectrode distance was 2.5 cm. The Coulbourn skin conductance coupler (V71-23) provided a constant 0.5 V across the electrodes. The analog signal was digitized at 10 Hz.

Participants continuously rated the US expectancy with a custom-built dial [34] on a scale ranging from 0 (certainly no breathing occlusion) to 100 (certainly breathing occlusion). The generated analog signal was digitized and stored at 10 Hz.

### Procedure

The procedure has been described in detail elsewhere [1], but we will also summarize the main elements here.

After determining the participant's maximal postexpiratory breath holding time (BHT), the experimenter attached the electrodes and explained how to use the mouthpiece and the breathing circuit. Participants were fitted with the mouthpiece and put on the noseclip, Next, a 10 min resting baseline of ECG was recorded. After this, the experimenter instructed the participant on how to use the US-expectancy dial. Following a startle habituation phase in which participants received 12 acoustic startle probes (10 s between probes), they went through one pre-exposure trial, 6 acquisition trials and 6 extinction trials. The pre-exposure trial consisted of: 25 s baseline, CS (8 s), and an ISI of 22 s. For the paired group, acquisition trials consisted of baseline (25 s), CS (8 s), US (40% of BHT), and ISI (27–30 s). The unpaired group received the following sequence during acquisition trials: baseline (25 s), CS (8 s), ISI (27–30 s), and US (40% of BHT). Extinction trials were never reinforced with a US and consisted of baseline (25 s), CS (8 s), and ISI (27–30 s+40% of the participant's BHT) for both groups. Startle probes were administered in each trial at random times between 5–7 s after CS onset, between 6 s after US onset and 2 s before US offset, and 21–23 s following the start of the ISI.

### Data Reduction and Analyses

Offline calculation of HRV (ECG) was performed using ARTiiFACT [35]. First, interbeat intervals (IBI) from the baseline ECG recordings were extracted. Artifacts were detected via an individually calculated distribution-related threshold criterion, were deleted, and values were estimated via linear interpolation of neighboring IBIs (for details see [35]). The time domain index of HRV used in our analyses was the root mean square differences of successive IBIs (RMSSD), a time domain measure of HRV that closely reflects parasympathetic influences on heart rate [17], [36]–[38].

EMG and EDA signals were treated offline with psychophysiological analysis software (PSPHA) [39]. For EMG startle blink, this software calculated a baseline for each 0–20 ms window following probe onset and subtracted this from the peak value detected in the subsequent 21–175 ms window. These responses were averaged for each subsequent pair of acquisition and extinction trials, leading to startle data for each person for 3 acquisition, and 3 extinction blocks. CS and ISI startle responses from acquisition and extinction were subsequently T-transformed within persons.

Electrodermal responses (skin conductance response, SCR) were calculated by subtracting the mean skin conductance level (SCL) during 1 s prior to CS onset from the maximum SCL during 6 s following CS onset. The responses were averaged for each subsequent pair of acquisition and extinction trials, leading to SCR data for each person for 1 pre-exposure, 3 acquisition, and 3 extinction blocks. These data were log transformed, Log10 (SCR+1), in order to obtain a normal distribution.

US-expectancy dial ratings for the 8 s during the CS presentation were also averaged across two subsequent trials within a phase, resulting in a mean rating for 3 acquisition and 3 extinction blocks.

Data from acquisition and extinction were tested separately in mixed model ANOVA designs. Each analysis included RMSSD at baseline as a continuous interindividual predictor variable and Group (paired/unpaired) and Block (1–3) as categorical independent variables. For startle EMG, an additional factor in the design was Probe (CS/ISI). Only Block and Probe were within subject variables. To allow displaying and further testing interaction effects involving RMSSD, we applied a median split of RMSSD, leading to 4 groups: a paired, low RMSSD group (n = 13, 3 males), an unpaired low RMSSD group (n = 16, 2 males), a paired high RMSSD group (n = 16, 2 males) and an unpaired, high RMSSD group (n = 12, 3 males). These pre-planned contrasts were tested using directional (1 tailed) t-tests consistent with the experimental hypotheses.

Alpha was set at .05. Greenhouse-Geisser corrections were applied where appropriate. Uncorrected degrees of freedom and corrected *p*s will be reported together with η^2^. Statistical analyses for all measures were accomplished with Statistica 8.

## Results

### RMSSD

The mean RMSSD during the 10 min baseline recording prior to the conditioning procedure was 42.50 (*SD* = 22.40; *range* = 8.40–108.07); gender differences were not significant (RMSSD of men: *M* = 47.50, *SD* = 28.85, *N* = 10, *range* = 13.94–108.07; RMSSD of women: *M* = 41.44, *SD* = 21.01, *range* = 8.40–106.03, *N* = 47; *t*(55) = −.77, *p* = .16). As expected, mean RMSSD values fell within the range of a healthy normal population [40].

### Startle EMG

#### Acquisition

**Figure 1:**
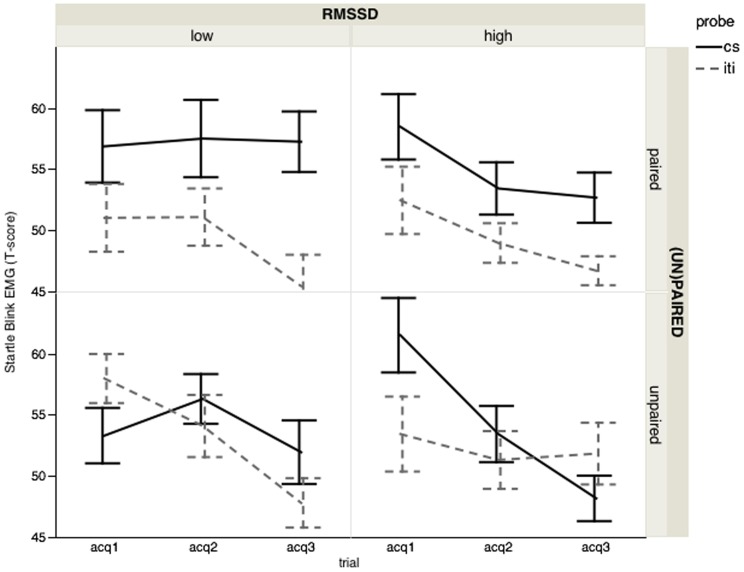
Startle blink responses during acquisition. Startle blink responses (T scores) of the paired and unpaired group during the CS load and the interstimulus interval (ISI) for the 3 acquisition blocks per RMSSD category (low – high). Only participants with high RMSSD in the unpaired group showed a decreasing linear trend in startle responding during the CS, suggesting more successful safety learning in the high compared to the low RMSSD unpaired group.

Follow-up comparisons of the significant Group×Probe interaction, *F*(1,53) = 4.89, *p*<.04, *η^2^* = .08, confirmed that only the paired group showed an enhanced startle response to the CS relative to the ISI during acquisition (paired: *F*(1,53) = 19.16, *p*<.01; unpaired: *F*(1,53) = 0.76, *p* = .39). However, this effect changed across acquisition blocks and was significantly modulated by interindividual differences in RMSSD, as evident from the Group×Probe×Block×RMSSD interaction *F*(2, 106) = 3.80, *p*<.03, *η^2^* = .07, *ε* = .94, see Figure 1. Follow-up analyses within each level of group showed that only in the unpaired group, RMSSD was a significant predictor of how CS-ISI differences changed across acquisition blocks (Probe×Block×RMSSD for the unpaired: *F*(2, 52) = 6.90, *p*<.01, *η^2^* = .21, *ε* = .98; for the paired: *F*(2, 52) = 0.23, *p* = .80, *η^2^* = .009, *ε* = .89). In the unpaired condition (Figure 1, Table 1), only participants with high RMSSD showed a decreasing linear trend in startle responding during the CS (unpaired low RMSSD: *t*(53) = 1.11, *p* = .46; unpaired high RMSSD: *t*(53) = 3.40, *p*<.001), suggesting more successful safety learning in the high compared to the low RMSSD unpaired group.

**Table 1:** Mean (standard deviation) of startle blink EMGs per phase, per experimental group, per RMSSD category, per probe and per block.

	Acquisition											
	Paired group						Unpaired group					
	Low RMSSD			High RMSSD			Low RMSSD			High RMSSD		
	block 1	block 2	block 3	block 1	block 2	block 3	block 1	block 2	block 3	block 1	block 2	block 3
CS	58.93 (2.56)	58.53 (2.24)	55.04 (2.30)	56.59 (2.65)	51.45 (2.32)	54.05 (2.38)	51.16 (2.75)	56.70 (2.40)	52.31 (2.47)	62.48 (2.65)	54.33 (2.32)	49.06 (2.38)
ISI	50.33 (2.55)	51.09 (2.12)	44.73 (1.99)	53.49 (2.64)	48.49 (2.20)	47.72 (2.06)	59.56 (2.74)	56.11 (2.28)	48.48 (2.14)	52.90 (2.64)	50.24 (2.20)	50.54 (2.06)

	Extinction											
	Paired group						Unpaired group					
	Low RMSSD			High RMSSD			Low RMSSD			High RMSSD		
	block 1	block 2	block 3	block 1	block 2	block 3	block 1	block 2	block 3	block 1	block 2	block 3
CS	51.82 (2.05)	48.44 (1.79)	49.50 (1.88)	50.55 (2.13)	48.37 (1.85)	45.57 (1.95)	47.43 (2.21)	43.18 (1.92)	46.45 (2.02)	50.82 (2.13)	47.17 (1.85)	46.64 (1.95)
ISI	44.71 (2.03)	42.39 (1.61)	44.54 (1.55)	44.64 (2.10)	43.19 (1.66)	42.08 (1.60)	45.79 (2.18)	45.67 (1.73)	42.13 (1.66)	45.72 (2.10)	44.71 (1.66)	45.09 (1.60)

#### Extinction

Similar to the effects observed during acquisition, both a Group×Probe interaction, *F*(1,52) = 5.27, *p*<.03, *η^2^* = .09, and a significant Group×Probe×Block×RMSSD interaction *F*(2, 104) = 3.09, *p*<.05, *η^2^* = .06, *ε* = .98, were present during extinction.

**Figure 2:**
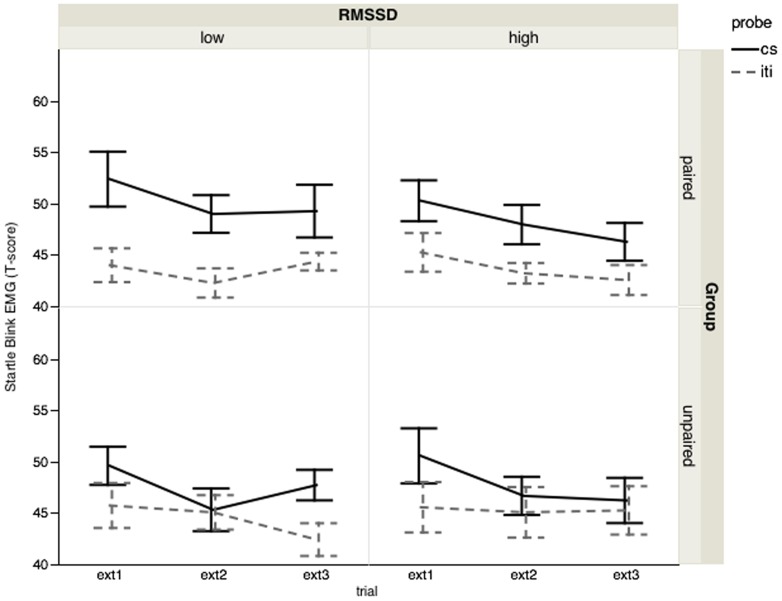
Startle blink responses during extinction. Startle blink responses (T scores) of the paired and unpaired group during the CS load and the interstimulus interval (ISI) for the 3 extinction blocks per RMSSD category (low – high). The linear decreasing trend for the CS was only significant in the high RMSSD paired group, but not in the low RMSSD paired group, suggesting better extinction in the former.

Figure 2 and Table 1 display extinction data for the paired group and suggest that extinction is more pronounced for participants with the highest compared to the lowest RMSSD (median split). The linear decreasing trend for the CS was significant in the high RMSSD group, *t*(52) = 1.85, *p* = .035, whereas it was not in the low RMSSD group, *t*(52) = 0.89, *p* = .19.

### Skin Conductance

#### Acquisition

**Figure 3:**
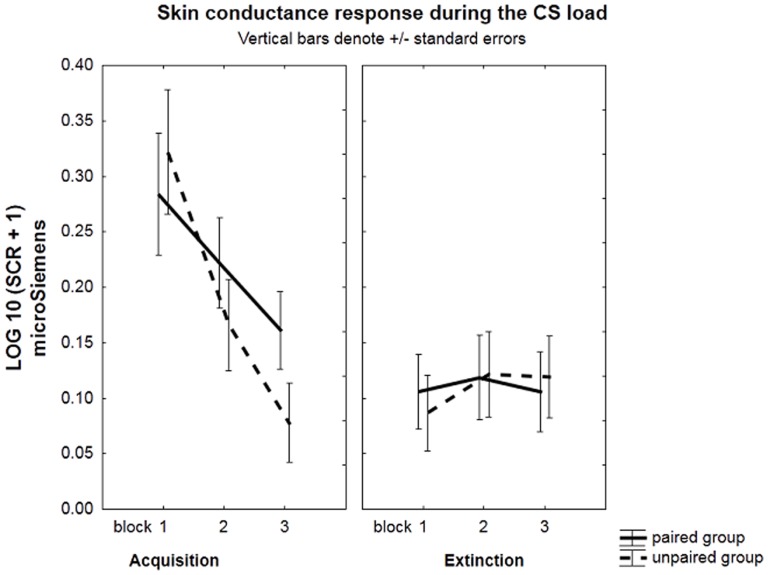
Skin conductance responses (SCR). Skin conductance responses (Log microSiemens) of the paired and unpaired group during the CS load for the 3 acquisition blocks and 3 extinction blocks. A much stronger decrease in SCRs from early to late acquisition was observed in the unpaired than in the paired group.

No significant effects were observed involving RMSSD during acquisition (Group×Probe×Block×RMSSD interaction, *F*(2, 106) = 0.16, *p* = .85, *η^2^* = .003. A marginally significant Group×Block interaction during acquisition, *F*(2, 108) = 2.8, *p* = .06, *η^2^* = .05, *ε* = .79, supported a learning effect. Further testing of this interaction indicated a much stronger decrease in SCRs from early to late acquisition for the unpaired (*t*(54) = 5.17, *p*<.001) than for the paired group (*t*(54) = 2.58, *p* = .005). See Figure 3.

#### Extinction

We did not found any significant effect involving RMSSD during extinction (Group×Probe×Block×RMSSD interaction, *F*(2, 106) = 0.16, *p* = .43, η^2^ = .002, *ε* = .99.

### US-expectancy

#### Acquisition

No significant effects involving RMSSD were present during acquisition or extinction (Group×Probe×Block×RMSSD interaction, for acquisition *F*(2, 106) = 0.51, *p* = .60, *η^2^* = .009, *ε* = .82; for extinction: *F*(2, 106) = 1.66, *p* = .20, *η^2^* = .03, *ε* = .99).

**Figure 4:**
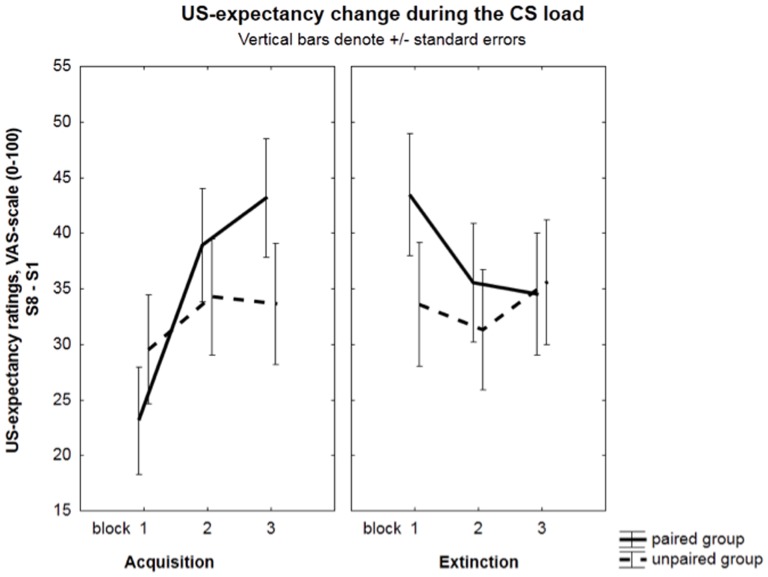
US-expectancy ratings. US-expectancy changes during the CS load of the paired and unpaired group for the 3 acquisition blocks and 3 extinction blocks. During acquisition, a linear increase in US expectancy ratings to the CS over blocks was only present in the paired group but not in the unpaired group.

However, a Group×Block interaction during acquisition, *F*(2, 110) = 3.15, *p* = .05, *η^2^* = .05, *ε* = .82, indicated that a linear increase in US expectancy ratings to the CS over blocks was only present in the paired group (*t*(55) = 3.70, *p*<.001) and not in the unpaired group (*t*(55) = 0.74, *p* = .23). See Figure 4.

#### Extinction

There were no significant effects, except for a marginally significant Group×Block interaction: *F*(2, 110) = 2.57, *p* = .08, *η^2^* = .04, *ε* = .99. Further testing of this interaction indicated a linear decrease in US expectancy ratings to the CS over blocks in the paired group (*t*(55) = 2.73, *p*<.001), but not in the unpaired group (*t*(55) = 0.58, *p* = 0.28).

## Discussion

The aim of the current study was to examine the relationship between vagally mediated HRV and fear inhibition in a between subject fear conditioning paradigm. In the paired group, a slight sensation of breathlessness (interoceptive CS) was consistently followed by a short-lasting suffocation experience (interoceptive US, complete breathing obstruction), while the same CS was never followed by the US in the unpaired group. Based on the neurovisceral integration model [11], we hypothesized that persons with higher levels of vagally mediated HRV would perform better in learning processes that involve fear inhibition. More specifically, we hypothesized that (1) higher resting HRV would be associated with more successful safety learning to the CS during acquisition in the unpaired group, and that (2) higher levels of HRV would be related to better fear extinction in the paired group. Our startle EMG data support both hypotheses.

The findings on safety learning during acquisition confirmed our hypothesis that HRV at rest modulates safety learning to the CS. In the unpaired group, the CS announced a relatively safe period, because it was never directly followed by the US. Our findings show that only participants with higher resting levels of HRV seem to learn this in terms of covert defense motor preparation, as reflected in their decreasing startle eye blink response during the CS from early to late acquisition.

Also our extinction findings support the idea that vagally mediated HRV is related to how easily inhibitory learning processes take place. An overall higher-order interaction for the startle data indicated that cardiac vagal outflow as measured by RMSSD prior to the conditioning procedure significantly explained some of the variance in extinction learning in the present experiment. Visualizing and further testing this interaction by means of a median split in RMSSD showed that, consistent with our hypothesis, the startle EMG response during the CS decreased more strongly in participants with a higher RMSSD compared to those with a lower RMSSD.

The present data add to findings from other studies that have already documented a negative association between HRV at rest and startle responding to non-threatening cues. For example, in an affective picture paradigm, Ruiz-Padial and colleagues [28] observed that persons with low resting HRV showed fear potentiated startle responses not only to negative, but also to neutral and positive pictures. Recently, these results have been replicated using a similar picture viewing paradigm [41]. In another recent study using the NPU threat paradigm [42], it was found that participants with a low HRV failed to inhibit defensive responding (startle reflex) particularly in conditions where the threat was unpredictable [29]. Taken together, these and our findings add to the idea that lower levels of resting HRV are associated with a general failure to inhibit defensive motor preparation to non-threatening cues. As such, they support the idea that vagally mediated HRV reflects the capacity of prefrontal vagal pathways to inhibit defensive responding [10]–[12].

A novel aspect of the present findings is that HRV at rest is related to inhibitory *learning processes* that have been suggested to play a role in the etiology and maintenance of pathological fear [3], [43]. One such learning process is extinction, which seems harder to establish in anxiety patients [44] and in non-clinically anxious persons [45]. During extinction, fear memories are not being erased, but a new memory is formed that can inhibit fear responding in a context-dependent way [46], [47]. More specifically, GABAergic intercalated cells (ITC) within the amygdala have been found to inhibit the central nucleus of the amygdala in the generation of fear responses, and the mPFC has excitatory connections to those ITC cells within the amgydala [40], [48]. Importantly, a similar pathway has recently been suggested to be associated with vagus nerve stimulation in a rodent model of fear extinction [49]. Our data suggest that extinction training is impaired in persons who have a generally reduced capacity of those mPFC inhibitory pathways to the amygdala, as reflected by their low levels of vagally mediated HRV at rest. Therefore, our findings suggest that the prefrontal vagal inhibitory pathways described by the neurovisceral integration model may to some extent overlap with neurobiological circuitry underlying extinction. The exact nature of this overlap remains an open question, because mPFC activations are typically more apparent during recall of extinction (24 hours following the extinction training), rather than during extinction training [5], [50]–[54]. It can be speculated that significant mPFC activity during extinction training can be observed only in participants with high HRV at rest. Suggestive in that regard are the results of a recent study [55]. Although the latter authors did not observe an overall significant (de)-activation of the prefrontal cortex during fear extinction, regression analyses revealed that highly trait-anxious subjects exhibited reduced dACC-activation.

Another inhibitory learning process is safety learning during acquisition. While the neurobiology of fear extinction has been extensively studied, less research has been performed to unravel the neurobiological substrate of safety learning in humans. Preliminary evidence is available however for the involvement of prefrontal cortical areas in safety learning as well [8], [9]. For example, it was demonstrated that activity in prefrontal cortex regions is positively correlated with fear ratings during threat/safety discrimination learning [8] and that anxious adults exhibit reduced activation in the ventromedial PFC when appraising threat [9]. Low levels of HRV and deficient safety learning have been documented apart from each other in anxiety disorder patients [56], [26], [3]. The present study adds to these findings by showing that both phenomena are related on the process level. In order to link up the present findings and hypothesis with the existing literature on safety learning, it would be interesting as well to test the association of a higher resting HRV with more successful safety learning in a standard differential paradigm with a CS+ (reinforced CS) and CS− (unreinforced) applied in the same participant.

Of all included measures only the fear-potentiated eyeblink reflex was modulated by HRV at rest. Another recent study [41] also showed that resting HRV was associated with affect-modulated characteristics of fear-potentiated startle, but not of skin conductance. The affective modulation of this fear-potentiated startle happens directly through activation of the amygdala via a simple brainstem and spinal cord pathway [57] and is therefore often considered to be a direct fear measure. Furthermore, since the amygdala is under tonic inhibition by the mPFC [58] of which vagal tone is thought to be a proxy, modulation of the eyeblink reflex is in this context of extreme interest. It fosters the hypothesis that HRV at rest might be a relevant predictor of subcortical, ‘hard-wired’ defensive responding.

The present findings may have important clinical implications. Because successful inhibitory learning seems to depend on the inhibitory strength of prefrontal vagal pathways, it may be useful for some patients to strengthen these prefrontal pathways, e.g., prior to entering an exposure treatment, or prior to entering situations with a great risk of traumatic events. It is yet unclear which procedures could establish this, but potentially effective candidates may include interventions that are known to induce increases in vagally mediated HRV at rest: mindfulness training [59], relaxation training [60], increasing physical fitness [61], dietary supplements of omega-3 fatty acids [62] and fish [63]. More directly, applying high-frequency repetitive transcranial magnetic stimulation (HF-rTMS) above prefrontal areas prior to exposure therapy might augment prefrontal inhibitory control during exposure. For example, a study of Baeken et al. [64] demonstrated that right HF-rTMS above the dorsolateral PFC attenuated right amygdala processing of negatively valenced emotional stimuli in healthy women.

The present study suffers from some important limitations that should be addressed in future studies. The strongest limitation may be the sample size, which is on the small side to reliably study interindividual difference variables. Whereas our main hypotheses were supported, low power may have prevented finding additional effects. Clearly replication with a larger sample is justified. Another limitation of this study is the lack of respiratory data. It has been demonstrated that breathing behavior under certain conditions may affect cardiac vagal tone [65]. However numerous prior studies of HRV and inhibitory processes have not found respiration to be crucial for these associations [28], [20]. Nonetheless, future studies should include measures of respiration to further verify the lack of association of respiration with the HRV effects in studies of inhibition. A third limitation is that our participants were mainly female (47 out of 57 participants) and that we did not collect any data on those women's menstrual cycle, despite recent studies having demonstrated the importance of menstrual cycle on fear inhibition processes [66]–[68]. Future studies should strive for a more equal distribution of both genders and should control for menstrual cycle effects in women.

In summary, we found an association between resting vagally mediated HRV and inhibitory learning. Persons with lower levels of HRV seem characterized by sustained anxiety and deficient safety learning. These results are in support of the neurovisceral integration model [11] that considers resting HRV as a proxy of medial prefrontal network activity underlying emotional regulation.
